# Exploring Intergenerational Communication on Social Media Group Chats as a Cancer Prevention Intervention Opportunity Among Vietnamese American Families: Qualitative Study

**DOI:** 10.2196/35601

**Published:** 2023-02-15

**Authors:** Huong Thien Duong, Suellen Hopfer

**Affiliations:** 1 Department of Health, Society, & Behavior Program in Public Health University of California, Irvine Irvine, CA United States

**Keywords:** cancer prevention, Vietnamese, family communication, intervention, colorectal cancer, human papillomavirus vaccine, HPV vaccine, Papanicolaou test, mobile phone

## Abstract

**Background:**

Families use social media group chats to connect with each other about daily life and to share information. Although cancer is not a frequent topic of conversation in family settings, the adoption of mobile technology in the family context presents a novel opportunity to promote cancer prevention information. To the best of our knowledge, few studies have used private social media group chats to promote cancer prevention information to family members.

**Objective:**

In this formative study, we investigated how family group chat platforms can be leveraged to encourage colorectal cancer screening, human papillomavirus vaccination, and cervical cancer screening among intergenerational Vietnamese American families. This study aimed to cocreate a family-based communication intervention for introducing cancer screening information in family group chats. We sought to understand family members’ motivations for using group chats, family dynamics and conversation patterns, and group chat experiences and cultural norms for interacting with family members.

**Methods:**

Overall, 20 audio-recorded and semistructured interviews were conducted with young Vietnamese adults. The study was conducted between August and October 2018. Participants were Vietnamese Americans; aged between 18 and 44 years; living in Orange County, California; had an existing family group chat; and expressed an interest in becoming family health advocates. Data were analyzed using a framework analysis.

**Results:**

In total, 13 (65%) of the 20 young adults reported having >1 group chat with their immediate and extended family. Preventive health was not a typical topic of family conversations, but food, family announcements, personal updates, humorous videos or photos, and current events were. Young adults expressed openness to initiating conversations with family members about cancer prevention; however, they also raised concerns that may influence family members’ receptivity to the messages. Themes that could potentially impact family members’ willingness to accept cancer prevention messages included family status and hierarchy, gender dynamics, relational closeness in the family, and source trust and credibility. These considerations may impact whether families will be open to receiving cancer screening information and acting on it. The participants also mentioned practical considerations for intervention and message design, which included the Vietnamese cultural conversation etiquette of *hỏi thăm*, respect for a physician’s recommendation, prevention versus symptom orientation, the family health advocate’s bilingual capacity, and the busy lives of family members. In response to exemplar messages, participants mentioned that they preferred to personalize template messages to accommodate conversational norms in their family group chats.

**Conclusions:**

The findings of this study inform the development of a social media intervention for increasing preventive cancer screening in Vietnamese American families.

## Introduction

### Background

The present communication landscape has inevitably shifted from in-person communication to technology-mediated contexts, which are now facilitated through social media messaging apps [1]. In the past decade, social media messaging platforms have been introduced into the family context, which has increased connectivity among family members [2]. This increased technology-mediated connectivity has social implications for family dynamics and communication [3,4].

Early forms of information and communication technologies focused on personal mobile communication, allowing a *narrow reach* (phone calls and SMS text messaging) between close friends or family on a one-on-one basis and a *wide reach* (using Twitter, Instagram, or Facebook) with distant acquaintances or strangers [4]. Now, group chat technology engages *middle-reach* audiences, which include immediate and extended family members [4]. Group chat apps have been studied to better understand family characteristics, personality, social support, frequency of use, and managing caregiving of family members [5-7]. To our knowledge, few studies have sought to understand how private group chats can be leveraged for disseminating health information to family members.

Cancer is the leading cause of death among Vietnamese Americans living in the United States [8,9]. Five-year age-adjusted human papillomavirus (HPV)-related cancers such as cervical cancer continue to be high in Vietnamese women in the United States (9.5 per 100,000) and colorectal cancer in both Vietnamese men and women (47.8 and 30.7 per 100,000, respectively) [8,10]. The high rates are due to a lack of early prevention behavior. Cervical cancer prevention measures include primary prevention measures (HPV vaccine) and secondary prevention measures (Papanicolaou test) [11,12]. Furthermore, for colorectal cancer, secondary prevention measures or early detection tools include colonoscopy and the fecal immunochemical test [11,13].

### Vietnamese Family Structure and Technology Use

Intergenerational communication between adult children and their older adult parents has increased the likelihood of colorectal cancer and hepatitis B screening in older adults [14,15]. However, existing studies lack the inclusion of communication with extended family networks and younger adults in mediated contexts. Vietnamese families value tight-knit structures, often including a complex network of extended family members such as aunts, uncles, cousins, and grandparents [16]. Vietnamese families experienced acculturation after resettling in the United States after the Vietnam war, causing discordance in relationships between the younger and older generations [17]. Despite this, family obligations, involvement, and values are still seen as important among young adult family members [18].

Families are increasingly using social media platforms to communicate with one another [19,20]. Minority groups, particularly Asian Americans, have been early adopters of smartphone technology to connect with family and friends [21]. The use of mediated platforms on smartphone technology presents an opportunity for prevention by introducing health topics into family conversations [22,23]. Smartphone apps such as WeChat, Viber, Facebook Messenger, and Kakao are popular apps used by many Asian American groups because they allow space for connecting on life events and health issues as well [4,19,24]. Research among caregivers of Vietnamese family members with dementia shows that they frequently use social media platforms, affording space for dementia education [19]. Particularly during the COVID-19 pandemic, the use of group chats to communicate about health became even more normalized, as this was a form of communication used to share information and provide social support to family members [25,26]. Using private group chat settings on smartphone-based apps allows smaller social networks such as families to foster more intimate conversations, which is an ideal setting for inserting cancer prevention information.

### Theories Guiding the Study

The Uses and Gratifications Theory (UGT) guided our research efforts to understand the motivations for family group chats [27]. Literature suggests that families tend to lean toward communication privacy in social media contexts; however, recent studies also show that family members use social media to share information with family members and cultivate greater openness [28,29]. UGT focuses on how people use media and their motivations for using specific channels [27]. UGT guided our understanding of why family members use their group chats, the types of preferred messaging platforms, and when they share information with their family members.

Given the family focus of these group chats, we also used the family communication pattern theory to guide our research efforts. The family communication patterns theory recognizes the importance of exploring how family dynamics and relationships impact communication patterns. According to this theory, intergenerational communication patterns among families are described as either conversation oriented or conformity oriented [30]. We sought to understand how family dynamics and communication norms may act as barriers and facilitators to conversations about health in the context of family group chats.

Finally, the principle of cultural grounding also directed our research. Recognizing the important role that culture plays in health, cultural grounding involves grounding the intervention development process and content in the experiences and expressions of the participants [31]. This entails having participants play an active role in cocreating culturally relevant material [31]. The principle of cultural grounding has been applied to intervention design in other contexts for school-based drug prevention programs, immigrant or rural health settings, and clinical trial participation promotion [32,33]. For this study, cultural grounding guided our intervention design by having Vietnamese young adults actively provide feedback on how, when, and which cancer prevention messages should be introduced into group chat conversations.

### Objectives

The purpose of this study was to understand (1) the topics that families talk about and share on their family group chats, (2) family members’ openness to cancer prevention conversations in the group chat context, and (3) how to introduce the topic of cancer prevention into family group chats to normalize the topic as a family conversation and increase its acceptability. We also sought to assess the feasibility of implementing an intervention to initiate conversations regarding colorectal and cervical cancer screening in group chat contexts among Vietnamese families.

## Methods

### Sampling Method

We conducted 20 semistructured interviews with young adults who self-identified as Vietnamese; were aged between 18 and 44 years; lived in Orange County, California; had an existing family group chat; and expressed an interest in becoming family health advocates (FHAs). The interviews lasted between 30 and 45 minutes and were conducted in person between August and October 2018. Orange County presently houses the largest Vietnamese ethnic enclave in America with approximately 200,000 Vietnamese residents, which comprises >33% of the Asian community in the county [34]. We used convenience sampling to recruit participants from local churches, youth groups, and students from university departments. The participants completed an interest form and were then contacted to confirm their eligibility.

### Ethics Approval

Institutional Review Board approval (HS# 2018-4454) was obtained from the University of California Irvine Institutional Review Board before the start of the study. Participants who displayed an interest in participating in the study were given the study information sheet to review beforehand. In addition, before the start of each interview, the interviewer verbally reviewed the study details, risks, and benefits with the participants. The participants provided verbal consent to participate in the study. The interviews lasted for approximately 1 hour and were audio recorded for accuracy purposes. The names were replaced with pseudonyms to protect participant identity. Each participant received US $50 as compensation for their time.

### Interview Guide

The interview guide questions focused on (1) family members’ motivations to use group chats, (2) family dynamics and conversation patterns, and (3) group chat experiences and cultural norms for interacting with family members. The questions also explored typical topics of conversations on family group chats, who participated in the group chat, what prompted information sharing in the group chat, and whether health was ever discussed.

The questionnaire focused on the frequency of group chat conversations, openness between children and older family members, and family group chat dynamics. The participants described typical communication patterns among family members on the group chat, their sense of the older adults’ openness to receiving health information from young adults, and Vietnamese family structures or beliefs that may influence the communication dynamics in the chat.

Finally, the young adults provided feedback about existing evidence-based cancer prevention messages adapted from the Centers for Disease Control and Prevention; the American Cancer Society; and the Asian American Network for Cancer Awareness, Research, and Training. They were also asked to provide feedback on the effectiveness of 2 culturally tailored HPV vaccine videos for Vietnamese young adults that are part of a National Cancer Institute evidence-based cancer control program called *HPV Vaccine Decision Narratives* [35]. The participants ranked preferred messages for their group chats, provided feedback about the messages, and described how they might adapt the messages when sharing them in the group chats. This activity engaged participants in the process of co-designing culturally resonant cancer prevention messages.

### Data Analysis

Data from interviews with FHAs were analyzed using the framework analysis [36]. The data analysis process began with verbatim transcription, followed by data immersion, familiarization of the range of responses, and the development of a thematic framework. Familiarization began during the data collection phase as interviews were transcribed and interviewer memos were reviewed. Using an inductive approach, data were tagged, and descriptive labels were assigned using NVivo Pro 11 (QSR International). This step was followed by a priori deductive coding of sensitizing constructs [37], which described how the participants viewed themselves concerning their family members. Some of the examples included *age*, *kid*, *close*, *trust*, *language*, and *lack of time*. After the primary and secondary coding cycles, data were organized into higher-order themes for the thematic framework. The thematic framework was then developed and categorized into the following themes: current group chat characteristics, cultural or familial barriers, facilitators for introducing cancer prevention messages into the group chat, cultural considerations for message design, and responses to exemplar messages.

Two coders met weekly to discuss the coding process and identify common characteristics and differences between codes to ensure intercoder agreement [37]. The first author was Vietnamese American who offered her perspective on the interpretation of the data for meaningful themes. Through member checking, the second author strengthened the validity of the findings [38]. The purpose of including the first author was to interpret the themes that resonated with the intended audience: Vietnamese American families. Percent agreement (Cohen κ statistic) was not calculated during the coding process [39].

## Results

### Demographics

In total, 20 individuals participated in the interviews. The mean age of the participants was 21 (SD 1.2) years. Most (17/20, 85%) participants identified as women, were enrolled in college, and were US-born second-generation Vietnamese Americans (refer to Table 1 for demographics).

**Table 1:** Demographic results (N=20).

Demographics		Values
Age (years), mean (SD)		21.1 (1.2)
**Gender, n (%)**		
	Woman	17 (85)
	Man	3 (15)
**Immigration status, n (%)**		
	US born	16 (80)
	Immigrant	4 (20)
**Generation, n (%)**		
	First	2 (10)
	1.5	2 (10)
	Second	16 (80)
**Level of education, n (%)**		
	Currently enrolled in college	18 (90)
	College graduate	2 (10)
**Vietnamese language proficiency, n (%)**		
	Limited	6 (30)
	Intermediate	9 (45)
	Advanced	5 (25)
**Number of family group chats per person, n (%)**		
	1	7 (35)
	2	8 (40)
	3	5 (25)

### Group Chat Characteristics

#### Social Media Platforms

Most (13/20, 65%) participants maintained several group chats with their extended family members. Among those who had multiple group chats, participants often had separate chats with just their cousins, with immediate family (parents and siblings), and with extended family (aunts, uncles, cousins, grandparents, and immediate family). Table 2 provides a list of different platforms used by young adults. The majority (14/20, 70%) of participants favored using Facebook Messenger because most family members had a Facebook account and because it was the easiest platform to communicate on.

**Table 2:** Social media group chat platforms used by family members (N=20).

Group chat platform	Participant use, n (%)
Facebook messenger	14 (70)
iMessage	13 (65)
SMS texting app	4 (20)
Facebook group page	3 (15)
Viber	2 (10)
WhatsApp	1 (5)

#### Conversation Topics

Families used group chats to share announcements; updates on family trips; graphic interchange formats or short, animated photos; and humorous videos related to common experiences (Table 3). Neither health nor cancer prevention was a typical topic of conversation in family group chats.

**Table 3:** Conversation topics and frequency of the topic mentioned (N=20).

Conversation topic	Participants who mentioned the topic, n (%)
Family events and announcements (eg, planning family gatherings)	18 (90)
Sharing news articles (eg, local, national, or world news)	9 (45)
Sharing food information (eg, recipes, meals, and grocery sales)	8 (40)
Personal updates (eg, health, whereabouts, school, and accomplishments)	8 (40)
Common family experiences (eg, sharing jokes and sending vacation photos)	7 (35)
Sharing humorous images (eg, memes and GIFs^a^)	5 (25)
Encouraging and supportive messages (eg, studying for exams)	2 (10)

^a^GIF: graphic interchange format.

### Introducing Cancer Prevention Messages Into Family Group Chat Conversations

#### Overview

Vietnamese American young adults anticipated some level of acceptance of cancer prevention messages among family members. Many recognized smartphone app affordances, such as convenience and maintaining social connections. Using such a platform to communicate information about cancer would be convenient for the family members. Furthermore, because the message would come from young adults embedded in the family group chat, their encouragement might influence family members’ acceptance of messages. Despite these affordances, they voiced concerns that family dynamics should be considered when introducing cancer prevention messages into a family group chat setting. Family member status, family hierarchy, gender dynamics, cultural norms, relational closeness, trust, and credibility were factors thought to influence the acceptance of cancer prevention messages. Table 4 shows a summary of the themes, including receptivity to cancer prevention messages and practical considerations for message design.

**Table 4:** Summary of themes.

Theme		Theme description
**Receptivity to cancer prevention messages**		
	Family member status and family hierarchy	Hierarchy and rank within the family structure could positively and negatively influence family members’ receptiveness to cancer screening information.
	Gender dynamics	Gender dynamics were also discussed as a barrier to discussing gender-specific cancers (eg, cervical cancer) if the opposite gender were present in the group chat.
	Cultural norms	Vietnamese family cultural norms play a role in how comfortable family members feel with engaging in conversations about cancer prevention. Some participants perceived discussing cancer prevention as taboo and not culturally acceptable within the family setting. Age was also mentioned as a concern of receptivity (eg, if a younger family member recommended screening to an older family member).
	Family relational closeness	FHAs^a^ discussed how relational closeness influences openness to accepting cancer prevention messages in family group chats. Relational closeness was seen as both a potential facilitator and barrier, depending on their perceived closeness with family members.
	Source trust and credibility	FHAs mentioned that their family members would trust them as a source of credibility because they have a college education or are actively pursuing a career in the medical field.
**Practical considerations for intervention and message design**		
	Cultural conversation etiquette	Several FHAs mentioned the cultural etiquette and conversational norm of *hỏi thăm,* which is asking generally about one’s overall well-being before any other conversation topic emerges.
	Respect for authority: a physician’s recommendation	FHAs acknowledged that the older generation has respect for their physician’s recommendation and opinion, which could both encourage and discourage screening.
	Prevention vs symptom orientation	Many participants stated that their family members tended to be more symptom oriented rather than prevention oriented, which presents another challenge for communicating prevention information to family members. Many FHAs mentioned that their families only take action when they feel “something is not right.”
	Vietnamese bilingual capacity	All participants (including intermediate and advanced speakers) described language as a barrier to communicating with older family members. They were prepared to use Google Translate and other workarounds (eg, involving parents and siblings) to translate for older family members.
	Busy lives and sustaining family cancer prevention conversations	Participants recognized time restrictions and busy schedules as barriers to engaging family members with cancer prevention information. FHAs mentioned that some family members may be more responsive than others given the time restrictions.

^a^FHA: family health advocate***.***

#### Family Member Status and Family Hierarchy

Several participants discussed how the family member status of the person introducing cancer prevention messages into family discussions played an important role. Hierarchy within the family structure could potentially influence family members’ receptiveness to cancer screening information. The typical Vietnamese family dynamic is patriarchal and embedded in values such as respect for older adults. Hieu, an 18-year-old young adult man, expressed apprehension about his family potentially being unwilling to listen to him:

I don’t know if it’s just my family or the entire community but...I’m eighteen but [I’m seen] as a kid in their eyes and...they would just dismiss [my messages] because this is a boy crying wolf type-of-thing...

For this participant, not being heard and his opinions not mattering to the older adults was a major concern. Tina, a 23-year-old woman, expressed similar concerns. She said the following:

...the older adults don’t really care about the things that the young people say to them.

Despite these concerns, young adults were still willing to be FHAs for their family members.

Although family hierarchy was perceived as a barrier to communication by some, it was perceived as a facilitator by others. Michael, a 22-year-old man, said in his response:

I’m the oldest cousin so I’m closest to the older generation than anybody else in the family. I have the best connection with them. Any information that I share would be the most well-received.

In Michael’s case, he perceived his age as a determining factor in his relationship with older family members. Other participants expressed that their family structures were not traditional and that their families were open to discussing cancer prevention between the older and younger generations.

#### Gender Dynamics

The participants expressed that the gender of the family member introducing cancer prevention was important when its incidence was sex specific, for example, in the case of cervical cancer prevention and Papanicolaou smear screening. Lily, a 21-year-old woman participant, voiced that:

I think especially among the Vietnamese older men, discussing [cervical cancer topics] can be uncomfortable and they might shut down

the conversation

Gender dynamics were also discussed as a barrier because of the potential awkwardness of discussing cervical cancer and Papanicolaou screening if the FHA advocating cancer screening was a man. Michael, a young adult who was interviewed, said in his reply:

Since I’m man, [Papanicolaou screening] is not the most relatable so I guess it’s awkward for me to send it out as well as on the receiving side

women family members

Although it is important to educate both men and women about HPV-related cancers, the gendered nature of certain cancers and screening tests may be difficult to discuss in the context of a mixed-gender family group chat.

#### Cultural Norms

Vietnamese family cultural norms play a role in how comfortable family members feel with engaging in conversations about cancer prevention. Some participants perceived discussing cancer prevention as taboo and culturally not acceptable within the family setting. Linh, a 21-year-old woman, said the following:

Culturally speaking, it’s not for everybody [in my family]. It’s stigma to talk about [cancer prevention]...It would probably take a couple of tries. As someone younger than them, it’s hard to discuss these

cancer topics

Linh mentioned how cancer is a stigmatized topic of conversation and how age could present challenges for introducing cancer prevention in conversations. In addition to cultural norms, relational closeness in Vietnamese families also plays a role in the acceptance of cancer prevention messages.

#### Family Relational Closeness

FHAs discussed how relational closeness may influence openness to accepting cancer prevention messages in family group chats. Relational closeness was perceived as both a potential facilitator and a barrier, depending on relationships with family members. Paula, a 21-year-old woman participant, said:

For my parents, since we’re closer, it’s easier to talk to them...so if there’s anything that’s bugging them, they would share it with me. For my cousins, it works the same way as my parents, but I don’t know how my aunt and uncle will take it.

Relational closeness affected whether the participants felt comfortable including their family members in a group chat setting and their hesitancy to initiate a conversation about cancer with these group chat members. Although some participants felt relational closeness was a difficult barrier to overcome, a select few mentioned that they felt confident that their family members would be more receptive to cancer prevention messages because of their perceived closeness within the relationship.

#### Source Trust and Credibility

Trust and credibility surfaced as facilitators for the acceptance of cancer prevention messages. Participants mentioned that their family members may trust cancer screening information from them because they are family. For example, Karen, a 21-year-old woman, responded:

Yeah, if it’s coming from me, they know that it’s important and they’ll actually listen rather than just a stranger telling them, “You should go get screened.”

Although participants mentioned that their role as a family member helps build trust, others also discussed the importance of credibility. Family members with medical or health science training were perceived as more credible. Allison, a 21-year-old participant, said:

[I think my family would trust the information] because it’s coming from me and regarding health...I’m going to school and I’m studying Pharmaceutical Sciences. They do know I am studying these things, so I do know certain things about health and informing them about these things.

In this case, personal trust in family members and credibility because of subject matter knowledge was important to consider when introducing cancer prevention messages into the group chat.

### Practical Considerations for Intervention and Message Design

The practical considerations for a group chat intervention included considering cultural conversational norms, respect for authority (the validity of a physician’s recommendation), family members being symptom oriented rather than prevention oriented, the necessity of bilingual messages, and the timing of the messages. Table 4 shows the summary of themes found when we asked FHAs to share their thoughts on family members’ receptivity to cancer prevention messages.

#### Cultural Conversation Etiquette

Some participants mentioned that it may be abrupt and awkward to introduce cancer prevention messages into family group chats without any pretext, as the topic is not typically discussed. FHAs suggested opening with messages expressing empathy and care for the health of family members instead. For example, Linh said:

First off, I know in our culture, we want to ask how someone is doing (hỏi thăm). That’s very important before you jump to a new topic. Ask how they are, how their health is [because] well-being is very important before you engage in anything.

Other participants offered similar sentiments that starting with overall well-being opens the conversation to introduce cancer topics.

#### Respect for Authority: a Physician’s Recommendation

Participants mentioned the importance of receiving cancer screening recommendations from medical authority figures. Respect for doctors’ opinions influences whether family members take suggestions for cancer screening from other family members seriously. One participant, Tammy, said:

I think [my family] would listen to me and they would consider it, but I don’t think they would ultimately do it until a doctor tells them to.

This theme indicates the need to build credibility for the advocated health behavior to effectively encourage family members to follow-up with cancer screening recommendations.

#### Prevention Versus Symptom Orientation

Participants stated that their family members tended to be more symptom oriented rather than prevention oriented, which presents another challenge for communicating prevention information to family members. Kelly, a 19-year-old participant, said:

I feel like my family might say that they don’t have time for [cancer screening] and...it’s not necessary because I think they’re living a lifestyle where they don’t feel they are susceptible to cancer so it might not be their top priority.

Another participant, Tina, expressed how her family also tended to be symptom oriented. She said:

Yeah, my dad’s side of the family is hard to communicate this information too because well, I try to be one step ahead of things like, “Oh! I got to see the doctor to do the preventive stuff,” but they are like, “We’ll take care of that when we get there or when we feel something.”

FHAs echoed the sentiment of taking action when “feeling something” or “something isn’t right,” which seemed to be a normal phenomenon in most families.

#### Vietnamese Bilingual Capacity

Participants described language as a barrier to communicating with older family members. Even intermediate and advanced speakers anticipated difficulties they might face when translating concepts from English to Vietnamese. Although 14 (70%) out of 20 participants expressed that they could speak and write both English and Vietnamese, there were still concerns about whether they would be able to communicate in Vietnamese with older members. For example, Jessica, a 21-year-old advanced Vietnamese speaker, said:

Even though I am an advanced speaker, there would be times where I say a sentence in Vietnamese, and I add an English word because that’s the first thing that comes [to] mind. I try my best not to do that, but it’s difficult. Sometimes I use google translate

but it’s not always right

Consequently, several participants mentioned that when designing messages, they needed to have readily translated material available to them.

Participants suggested workarounds for the anticipated language barrier with first-generation family members who spoke less English. Including other family members in the translation of concepts was a strategy offered by young adults. For example, Michael said:

For my grandparents, there is a language barrier...they prefer Vietnamese, but my aunt is there [to help] which is great.

FHAs were prepared to work around the problem with a variety of methods, which included using Google Translate, asking for a researcher’s help, or asking other Vietnamese-speaking family members to help facilitate conversation.

#### Busy Lives and Sustaining Family Cancer Prevention Conversations

Participants recognized time restrictions and busy schedules as barriers to engaging family members with cancer prevention information. FHAs mentioned that some family members may be more responsive than others given the time restrictions. Recommendations included strategically disseminating social media group chat messages in the evenings. For example, Tracy, a 21-year-old woman participant, said:

It would be best if I talked to them at night because everyone in my family is working late. I’m not sure how engaged my mom will be because she works until 8 or 9 p.m. When she comes back, she just wants to chill and not think about cancer. It would be best to talk to her on her day off and she’d be more responsive to me.

Other participants described similar predictions that since group chats are synchronous, participants could ignore or delay reading the messages. Long, a 21-year young adult man, said:

I think it might take 2-3 days for them to read it...but if it concerns their health, they’ll be inclined to do all they can to prevent it.

The short time to have a productive conversation was a recurring theme to consider in the intervention design.

### Responses to Example Messages

The concluding section of the interview asked participants to review existing messages about colorectal cancer and HPV-related cancers from publicly available text or social media messages, infographics, websites, and video examples adapted from the Centers for Disease Control and Prevention; the American Cancer Society; and the Asian American Network for Cancer Awareness, Research, and Training. Participants wanted to be able to tailor messages “in their own voice” because the messages were “too academic” or sounded “too much like an advertisement.” Relevance to family members, such as specific demographics, age, and gender tailoring, were also essential elements to be considered. Participants also expressed their wish for messages to include symptoms, be actionable and shorter. Table 5 shows message ranks by popularity, positive feedback, and constructive criticism to exemplar messages.

**Table 5:** Feedback to message examples.

Message example			Positive feedback	Constructive criticism
**Colorectal cancer messages**				
	“Did you know Asian Americans are at higher risk for colorectal cancer? Though the best test is the colonoscopy, you can get screened at home using the FIT^a^ test. Learn more here.”	Demographic tailoring for Asian Americans Open ended and poses a question Neutral message Feasible and specific actions Targets susceptibility Offers alternative option for screening		Vague language and not as informative Not a specific Asian subgroup Does not mention age at screening “Advertising” language Lacking statistics
	“Don’t ignore symptoms of colon cancer! If you are experiencing pain in the abdomen, blood in stool, body fatigue, and weight loss, talk to your doctor right away. Click here for more info.”	Emphasizes urgency Symptoms are informative		Off-putting or offensive to older adults Demanding tone and not genuine
	“If you are 50 or older, you need to be screened for colorectal cancer. Even if you feel healthy, make sure to talk to your doctor about getting screened. Click on this link for more information.”	Age tailored To the point		Not relevant to people aged <50 years Not detailed enough
	“No matter how old you are, there are ways to prevent colorectal cancer through diet, exercise, and not smoking tobacco. Click on this link for more information.”	Feeling healthy may not mean you are healthy Relevant to both young and older adults Neutral message Relevant to smokers in the family		Not as relevant or targeted “Advertising” language Broad symptoms that could be for any other disease (easy to ignore) Does not provide new information that is not already known
**HPV^b^-related cancer messages**				
	“The rate of cervical cancer among Vietnamese American women is 40% higher than Whites. Cervical cancer can be prevented by getting an HPV vaccine, visiting your doctor for a Papanicolaou test when recommended, and not smoking. Click on this link for information.”	Demographic is relatable Comparison statistics Actionable behaviors		Needs to be less formal in language Lengthy information
	“The HPV vaccination is not only for women! HPV vaccination is recommended for young men and women through age 18-26. Talk to your doctor about getting vaccinated. Click for more info.”	Inclusive of both women and men Addresses misconceptions about the HPV vaccine for women Age tailored		Sounds like a PSA^c^ or advertisement
	“Pap screening is necessary for cervical prevention even if you’ve already received the HPV vaccine. Pap screening is recommended for women 21 or older every three years. Click here for info.”	Emphasizes receiving screening even if vaccinated		Not engaging or interesting Not relevant to men Not as personal Needs more explanation between screening and vaccine
	“One of the most important things you can do to help prevent cervical cancer is to have regular screening tests starting at age 21 and repeat as recommended. Click on this link for more info!”	Highlights prevention Age relevance		Not enough information No hook General information and not as impactful Does not mention why it is important “Regular screening” does not imply urgency

^a^FIT: fecal immunochemical test.

^b^HPV: human papillomavirus.

^c^PSA: public service announcement.

## Discussion

### Principal Findings

The purpose of this study was to understand how to effectively engage Vietnamese family members with cancer prevention messages in group chats. The results provide insights into the development of a social media cancer prevention intervention for family contexts. Although the social media literature focuses on how media affects people’s mood and well-being, our study explored family members’ motivations for using media to identify opportunities for entry points of influence for promoting cancer screening [27]. Vietnamese families use their family group chats to stay up-to-date with family members’ daily lives, family gatherings, and food or to provide moral support to others in the family group. Family group chat conversations around the planning of family celebrations potentially provide an entry point of influence to introduce cancer prevention discussions. The timing of discussions at Tết (Vietnamese New Year) may be beneficial for introducing the importance of cancer screenings. The pandemic has brought renewed attention to vaccination and discussion of health in family group chats.

Although prior research has recognized that people use many social media platforms such as Twitter, Facebook, and Instagram [40,41], FHAs mentioned using several family group chats on a single platform. Most FHAs mentioned that they had established several family group chats, segregated by the nuclear family (mom, dad, and siblings), peers (cousins and siblings), and intergenerational extended family (grandparents, aunts, uncles, parents, and cousins). Given this phenomenon, topics such as HPV vaccination should be disseminated in a peer family group chat, whereas topics such as colorectal cancer screening should be promoted in an intergenerational group chat context.

Several social influence strategies to encourage cancer screening in group chat contexts could be applied based on the findings of our study. Segmenting audiences by age and family relationships is one strategy mentioned; however, we may also consider how typical topics of group chat conversations such as food, humorous “memes,” family announcements, or even family health history can function as a social influence entrée to connect and introduce cancer prevention [42,43].

Another strategy may include building information requests off preexisting entry points. For example, using typical group chat activities such as sharing family memories through pictures and reminding family members of cancer screening recommendations to stay healthy may be a way to integrate cancer information. Family group chats also offer the potential to apply a foot-in-the-door social influence strategy [44]. This strategy initially involves making a small request to a family member that is likely to yield a positive response, followed by a cancer screening request (ie, a slightly more demanding request). Communication accommodation theory [45] also suggests the importance of adapting cancer screening messages to personalize and adapt to the cultural context of Vietnamese families’ conversational norms, which may involve the timing of introducing the message, who the message sender is, or how the message is introduced.

Our study lays out a range of innovative approaches to introducing and normalizing the topic of cancer screening within mediated family conversation contexts. Several key factors must be considered for message design including family dynamics, culture, language, and conversational norms for family receptivity to messages. Our results show the importance of aligning interventions to match cultural norms for increasing cancer screening acceptance among Vietnamese people [31,46]. This sheds light on the group chats as a novel strategy for introducing and reinforcing cancer screening among family members. For example, having an “inside family member” vouch for acting on screening recommendations from the doctor has the potential to reinforce cancer screenings in an informal web-based setting [22].

In addition, as part of cultural tailoring, women need to introduce “woman” cancer screening messages (eg, Papanicolaou screening) to other women family members. Ensuring that the young adult family member introducing cancer screening is the elder grandchild or a student in the health field can also increase the likelihood of message acceptance by family members. Young adults also expressed the desire to self-tailor cancer screening messages. Cocreating and adapting messages to familiar formats aligns with the principle of cultural grounding and is more likely to resonate with the target audience [31]. For example, one study found that self-tailored arguments by parents about HPV vaccination were more persuasive than motivational interviewing in a clinical parent intervention [47]. Allowing young adults to self-tailor screening messages for their families and insert their motivations as justification may increase acceptance of receiving messages about cancer prevention.

Finally, young adults expressed interest, openness, and motivation to participate and facilitate an intervention with their family members despite potential challenges. Although expectations by the family include that health information is typically delivered by medical professionals in medical settings, receiving reinforcement messages from trusted family members is equally important [48]. Therefore, receiving cancer prevention messages from trusted family members offers another strategy to reinforce credibility and prevent cancer. Reinforcement messages can be key to moving individuals toward actionable behaviors [49].

### Limitations

Vietnamese young adults from Orange County, California, were interviewed to identify key factors to consider when introducing cancer screening messages as part of family group chats. Data saturation for understanding the influence of family dynamics on group chat conversations may be incomplete because our data reflect the family dynamics of our informants [50]. The data generated in this study reflect the experiences and thoughts of Vietnamese women, who were the majority in this study. The perspectives of Vietnamese men were less represented. Furthermore, the findings represent the perspectives of the young adult FHAs and not the entire family group. Future research should consider the older family members’ thoughts on messaging, accessibility, and acceptability. Finally, this study was conducted before the COVID-19 pandemic, which changed how families discuss health. This study generated important considerations for effectively introducing culturally grounded cancer screening messages into Vietnamese family group chats.

### Conclusions

The results of this study help to understand (1) the feasibility of developing a social media intervention among Vietnamese families and (2) family communication norms and cultural considerations for effective intervention design. Given the increased popularity of social media use for family communication, it is important to continue this line of research to understand how social media platforms and intervention designs can be leveraged to encourage preventive screening. Not only is it important to understand how these platforms are used, but it is also crucial to understand what motivates families to actively participate in group chats. Furthermore, understanding family dynamics, family conversation, and the role of Vietnamese family culture will advance our understanding of how to effectively communicate cancer prevention with Vietnamese families, improve health outcomes, and reduce late-stage cervical and colorectal cancer incidences among Vietnamese Americans.

## Abbreviations

FHA: family health advocate
HPV: human papillomavirus
UGT: Uses and Gratifications Theory
